# Oxidative Stresses and Mitochondrial Dysfunction in Age-Related Hearing Loss

**DOI:** 10.1155/2014/582849

**Published:** 2014-07-03

**Authors:** Chisato Fujimoto, Tatsuya Yamasoba

**Affiliations:** Department of Otolaryngology, Faculty of Medicine, The University of Tokyo, 7-3-1 Hongo, Bunkyo-ku, Tokyo 113-8655, Japan

## Abstract

Age-related hearing loss (ARHL), the progressive loss of hearing associated with aging, is the most common sensory disorder in the elderly population. The pathology of ARHL includes the hair cells of the organ of Corti, stria vascularis, and afferent spiral ganglion neurons as well as the central auditory pathways. Many studies have suggested that the accumulation of mitochondrial DNA damage, the production of reactive oxygen species, and decreased antioxidant function are associated with subsequent cochlear senescence in response to aging stress. Mitochondria play a crucial role in the induction of intrinsic apoptosis in cochlear cells. ARHL can be prevented in laboratory animals by certain interventions, such as caloric restriction and supplementation with antioxidants. In this review, we will focus on previous research concerning the role of the oxidative stress and mitochondrial dysfunction in the pathology of ARHL in both animal models and humans and introduce concepts that have recently emerged regarding the mechanisms of the development of ARHL.

## 1. Introduction

Oxidative stress represents an imbalance between the production of reactive oxygen species (ROS) and the detoxification of their reactive intermediates. ROS, such as hydroxyl radicals, superoxide anions, hydrogen peroxide, and singlet oxygen, are primarily generated by mitochondria in most mammalian cells and are generally regarded as the toxic side-products of cellular metabolism [1–3]. ROS are normally detoxified by a variety of antioxidant enzymatic scavengers, including superoxide dismutase (SOD), catalase, glutathione S-transferase (GST), and glutathione peroxidase (GPX) [4].

Mitochondria are a major site of ROS-induced oxidative damage [5, 6]. ROS generated by mitochondria are hypothesized to damage key mitochondrial components such as mitochondrial DNA (mtDNA), mitochondrial membranes, and respiratory chain proteins and nuclear DNA that affect mitochondrial function. mtDNA is a circular, closed, double-stranded molecule and is not protected by histones. Therefore, mtDNA is more susceptible to DNA insults in comparison with nuclear DNA. Most of mtDNA mutations are characterized by heteroplasmy, which is defined as the presence of more than one an organellar genome within a cell or tissue from a single individual. As the percentage of mutant alleles increases, the mitochondrial bioenergetic defect becomes more severe. The expression of disease depends on the percentage of mutant alleles.

It has been widely considered that aging is the process of accumulated oxidative damage caused by ROS [7, 8]. This damage accumulates over time, causing mitochondrial dysfunction and an associated decrease of energy production, and results in tissue dysfunction. ROS production increases with age and it is known that oxidative stress and associated mitochondrial dysfunction play an important role in aging and age-related diseases [1, 2].

Age-related hearing loss (ARHL), which is also called presbycusis, is the progressive loss of hearing associated with aging and is the most common sensory disorder in the elderly population [9–11]. ARHL afflicts approximately half of the people over 65 years of age in the United States [12]. The prevalence of the ARHL is expected to increase as the elderly population grows [9, 13, 14]. It has been proposed that ARHL is associated with many factors, including environmental, medical, and hereditary factors [12, 15]. So far, no effective treatment has been found for this age-related disorder.

Many studies have been conducted based on the assumption that age-related oxidative stress and mitochondrial dysfunction could be an underlying pathology of ARHL as well as other age-related diseases. In this review, we will focus on previous research concerning the role of the oxidative stress and mitochondrial dysfunction in the pathology of ARHL in both animal models and humans and introduce concepts that have recently emerged as potential mechanisms for the development of ARHL.

## 2. Pathological Findings in ARHL

Sound waves travel down the external ear canal and cause the tympanic membrane to vibrate. The ossicles in the middle ear link the vibrating tympanic membrane to the cochlea, the auditory end organ of the inner ear. The cochlea is filled with fluid that vibrates in response to the movement of the ossicles. The inner and outer sensory hair cells are located within a core component of the cochlea, the organ of Corti. When a sound pressure wave travels from the basal turn to the apical turn of the cochlea, the basilar membrane vibrates [16]. Displacement of stereocilia, the mechanosensing organelles of the hair cell, in association with the vibration of the basilar membrane, opens transduction ion channels, allowing entry of potassium ions from the endolymph produced by the stria vascularis. This transduction current then activates voltage-dependent calcium channels along the hair cell lateral wall and base [17]. The inner hair cells release the neurotransmitter glutamate to encode acoustic signals for the adjacent spiral ganglion neurons (SGNs), which are the primary auditory neurons [18].

Based on postmortem pathological analysis, ARHL in humans is generally classified into 3 types: sensory hearing loss (loss of sensory hair cells), neuronal hearing loss (loss of SGNs), and metabolic hearing loss (atrophy of the stria vascularis) [9, 19], although it is now well established that most cases of ARHL exhibit mixed pathological changes [9]. This idea is supported by the observation that the progressive loss of hair cells and SGNs leads to ARHL because these two cell types do not regenerate in mammals.

## 3. Candidate Genes for ARHL Associated with Oxidative Stress and Mitochondrial Dysfunction

Many genetic investigations of ARHL, such as genome-wide association studies and candidate-gene-based association studies, have been performed recently [20]. With regard to oxidative stress and mitochondrial function, several genes and loci have been proposed as a result of candidate-gene-based association studies, which are based on hypotheses about the relationship between specific known loci and phenotypes.

The superoxide dismutases (SODs), which catalyze the dismutation of superoxide into oxygen and hydrogen peroxide, are an important part of the antioxidant defense system against ROS. Recently, evidence from the London ARHL cohort suggested an effect of common superoxide dismutase 2 (SOD2, also known as manganese SOD or mitochondrial SOD) promoter variation, −38 C > G, on SOD2 promoter regulation and linked it to ARHL risk in men; however, this association was only suggestive due to a lack of replication [21].

The glutathione S-transferases (GSTs) catalyze the detoxification of electrophilic substrates by conjugation with reduced glutathione and participate in intracellular binding and transport of lipophilic substances. Decreased glutathione and GST activity levels cause an increase in susceptibility to cell damage. A previous study investigated the association between ARHL and genes related to oxidative stress using a large set of samples from two population groups, a general European group and a Finnish group [22]. Although an association between the polymorphisms of glutathione S-transferase, mu 1 (*GSTM1*) or glutathione S-transferase, theta 1 (*GSTT1*), and ARHL was not detected in the former population, there were significant associations between both genes and ARHL in the latter population.

Mitochondrial uncoupling proteins (UCPs), which are members of the larger family of mitochondrial anion carrier proteins, facilitate the transfer of anions from the inner to the outer mitochondrial membrane and the return transfer of protons from the outer to the inner mitochondrial membrane. UCPs reduce the mitochondrial membrane potential in mammalian cells. The main function of uncoupling protein 2 (UCP2) is the control of mitochondria-derived ROS [23].* UCP2* Ala55Val polymorphisms exhibited a significant association with ARHL in a Japanese population [24].

## 4. Deletions and Mutations of mtDNA in the Peripheral Auditory System of ARHL Patients

Acquired mtDNA defects have been proposed as important factors in aging. Increases in deletions, mutations, or both, in mtDNA have been reported in human temporal bone studies from ARHL patients in comparison with normal-hearing control tissues. A 4977-base pair deletion of mtDNA from celloidin-embedded temporal bone sections was significantly more frequent in cochlear tissue from ARHL patients in comparison to those with normal hearing [25]. Another study reported that quantitative analysis of the mtDNA in archival cochlear tissue samples revealed a mean common deletion level of 32 ± 14% in ARHL patients, in comparison with a level of 12 ± 2% in age-matched controls with normal hearing, and showed a significant correlation between the common deletion level and the severity of hearing loss [26]. Cytochrome c oxidase subunit 3 (COX3) expression was significantly diminished in SGNs from ARHL patients in comparison with age-matched normal-hearing individuals. In addition to the mtDNA common deletion, other deletions involving the mtDNA major arc contributed to the observed deficit in COX3 expression [27]. Mutations within the cytochrome c oxidase subunit 2 (*COX2*) gene in the spiral ganglion and membranous labyrinth from archival temporal bones occur more commonly in ARHL patients relative to controls [28].

## 5. Basic Research in Animals on the Role of Oxidative Stresses and Mitochondrial Dysfunction in ARHL

Although details of the aging process differ in various organisms, there is a common understanding that oxidative stress and mitochondrial dysfunction play a major part in aging. The auditory system is no exception and it is thought that oxidative damage caused by ROS and mitochondrial dysfunction plays a causal role in ARHL. The fast-aging senescence-accelerated mouse-prone 8 (SAMP8) strain that is a useful model for probing the effects of aging on biological processes displays premature hearing loss associated with strial, sensory, and neural degeneration [29]. The molecular mechanisms associated with premature ARHL in SAMP8 strain mice involve oxidative stress, altered levels of antioxidant enzymes, and decreased activity of complexes I, II, and IV, which lead to triggering of apoptotic cell death pathways.

In the organ of Corti of CBA/J mice, glutathione-conjugated proteins, markers of H_2_O_2_-mediated oxidation, were shown to begin to increase at 12 months, and 4-hydroxynonenal and 3-nitrotyrosine, products of hydroxyl radical and peroxynitrite action, respectively, were elevated by 18 months [30]. On the other hand, apoptosis-inducing factor and SOD2 were decreased by 18 months in the organ of Corti and SGNs [30]. Mice lacking superoxide dismutase 1 (*Sod1*) showed premature ARHL [31, 32]. Age-related cochlear hair cell loss was observed in* Sod1* knockout mice [32] and a reduced thickness of the stria vascularis and severe degeneration of SGNs were observed at middle age [31]. A previous study showed that increased GPX activity was observed in the stria vascularis and spiral ligament of the cochlea in aged Fischer 344 rats [33]. Two-month-old knockout mice with a targeted inactivating mutation of the gene coding for glutathione peroxidase 1 (*Gpx1*) showed a significant increase in hearing thresholds at high frequency [34]. Mice lacking senescence marker protein 30 (SMP30)/gluconolactonase (GNL), which are not able to synthesize vitamin C, showed a reduction of vitamin C in the inner ear, an increase of hearing thresholds, and loss of spiral ganglion cells, suggesting that depletion of vitamin C accelerates ARHL [35]. Oxidative stress induces the expression of BCL2-antagonist/killer 1 (*Bak*); the mitochondrial proapoptotic gene, in primary cochlear cells and Bak deficiency prevents apoptotic cell death [36]. C57BL/6J mice with a deletion of* Bak* exhibit reduced age-related apoptotic cell death of SGNs and hair cells in the cochlea and prevention of ARHL [36]. A mitochondrially targeted catalase transgene suppresses* Bak* expression in the cochlea, reduces cochlear cell death, and prevents ARHL [36]. Collectively, these findings indicate that age-related increases in ROS levels play an important role in the development of ARHL.

It has been shown that accumulation of mtDNA mutations leads to premature aging in mice expressing a proofreading-deficient version of the mtDNA polymerase g (POLG D257A mice), indicating a causal role of mtDNA mutations in mammalian aging [37, 38]. POLG D257A mice accumulate mitochondrial mutations more rapidly than wild-type mice. At 9-10 months old, POLG D257A mice showed a variety of premature aging phenotypes, including the early onset of ARHL. Histological findings in the cochlear basal turn confirmed that POLG D257A mice at the age of 9-10 months showed a severe loss of SGNs and hair cells and significant elevation in TUNEL-positive cells and cleaved caspase-3-positive cells in the cochlea [39].

Mitochondrial biogenesis and degradation are involved in mitochondrial turnover. In the SGNs of SAMP8 strain mice, mitochondrial biogenesis, characterized by the ratio of mtDNA/nuclear DNA and the activity of citrate synthase, was increased at younger ages and decreased in old age [29]. Age-related reductions of peroxisome proliferator-activated receptor c coactivator a (PGC-1a), one of the key regulators of mitochondrial biogenesis, might be an important factor for mitochondrial function in age-related diseases [40]. When it comes to mitochondrial function in the cochlea, the overexpression of PGC-1a with a consequent increase of nuclear respiratory factor 1 (NRF1) and mitochondrial transcription factor A (TFAM) caused a significant decrease in the accumulation of damaged mtDNA and the number of apoptotic cells in the strial marginal cells senescence model [41]. Autophagy is one of the major intracellular degradation pathways along with the ubiquitin-proteasome system [42]. Unnecessary cytoplasmic proteins and organelles are enclosed by the autophagosome and then delivered to the lysosome by autophagy. It has been reported that the SGNs of SAMP8 undergo autophagic stress with accumulation of lipofuscin inside these cells [29]. Downregulation of mitophagy, the selective removal of damaged and dysfunctional mitochondria by autophagosomes will cause abnormal mitochondrial morphological changes. Impairment of mitophagy might result in the formation of giant mitochondria, which have been characterized as having low ATP production, a loss of cristae structure, and a swollen morphology [43]. Accumulation of abnormally functioning and shaped mitochondria accelerates apoptosis [44], which merits further investigation in the cochlea.

## 6. Prevention and Retardation of ARHL by Supplementation or Caloric Restriction

Several studies have reported the effects of supplementation of antioxidants against ARHL. A cross-sectional and 5-year longitudinal study in Australia demonstrated that dietary vitamin A and vitamin E has a significant association with the prevalence of hearing loss, although dietary antioxidant intake did not increase the incidence of hearing loss [45]. Another cross-sectional study in Australia showed that higher carbohydrate, vitamin C, vitamin E, riboflavin, magnesium, and lycopene intakes were significantly associated with larger transiently evoked otoacoustic emission (TEOAE) amplitudes and better pure tone averages (PTAs) whereas higher cholesterol, fat, and retinol intakes were significantly associated with lower TEOAE amplitude and worse PTAs [46]. Another further cross-sectional study in the United States showed that higher intakes of beta-carotene, vitamin C, and magnesium were associated with better PTAs at both speech and high frequencies, and high intakes of beta-carotene or vitamin C combined with high magnesium compared with low intakes of both nutrients were significantly associated with better PTAs at high frequencies [47].

In animal studies, Fischer 344 rats given vitamin C, vitamin E, melatonin, or lazaroid had better auditory sensitivities and a trend for fewer mtDNA deletions in comparison with placebo subjects [48]. Fischer 344 rats of 18–20 months old supplemented orally for 6 months with lecithin, a polyunsaturated phosphatidylcholine (PCP) which has antioxidant effects, showed significantly better hearing sensitivities, higher mitochondrial membrane potentials, and reduced frequency of the common aging mtDNA deletion in the cochlear tissues compared with controls [49]. Aged dogs fed a high antioxidant diet for the last 3 years of their life showed less degeneration of the spiral ganglion cells and stria vascularis in comparison with dogs fed a control-diet [50]. In C57BL/6 mice, supplementation with vitamin C did not increase vitamin C levels in the cochlea or slow ARHL [35], but animals fed with a diet comprising 6 antioxidant agents (L-cysteine-glutathione mixed disulfide, ribose-cysteine, NW-nitro-L-arginine methyl ester, vitamin B12, folate, and ascorbic acid) showed significantly better auditory sensitivity [51]. When C57BL/6 mice were fed with a diet containing one of 17 antioxidant agents (acetyl-L-carnitine, alpha-lipoic acid, beta-carotene, carnosine, coenzyme Q10, curcumin, d-alpha-tocopherol, epigallocatechin gallate, gallic acid, lutein, lycopene, melatonin, N-acetyl-L-cysteine, proanthocyanidin, quercetin, resveratrol, and tannic acid), ARHL was nearly completely prevented by alpha-lipoic acid and coenzyme Q10 and partially by N-acetyl-L-cysteine, but not by other agents [36]. When CBA/J mice were fed with an antioxidant-enriched diet containing vitamin A, vitamin C, vitamin E, L-carnitine, and a-lipoic acid from 10 months through 24 months of age, the antioxidant capacity of the inner ear tissues was significantly increased, but the loss of hair cells and spiral ganglion cells and the magnitude of ARHL were not improved [52]. These studies show that the prevention and retardation of ARHL by supplementation with antioxidants can be influenced by many factors such as the type and dosage of antioxidant compounds, the timing and duration of the treatment, and the species and strains involved.

Caloric restriction (CR) extends the lifespan of various organisms including yeast, worms, flies, rodents and non-human primates. It has been reported that CR plays an important role in reducing age-related diseases such as cancer [53], protecting age-related mitochondrial dysfunction [54] and reducing mtDNA damage [55]. It has also been reported that CR can protect neurons against degeneration in animal models of neurodegenerative diseases, as well as promote neurogenesis and enhance synaptic plasticity [56]. The ability of CR to prevent cochlear pathology and ARHL has been extensively studied using laboratory animals [57]. C57BL/6 mice with CR by 15 months of age maintained normal hearing and showed no obvious cochlear degeneration and a significant reduction in the number of TUNEL-positive and cleaved caspase-3-positive cells in the spiral ganglion cells in comparison with controls [58]. Fischer 344 rats with CR to 70% of the control intake beginning at one month of age and then housed for 24-25 months showed significantly better hearing thresholds, reduced hair cell loss, and decreased mtDNA common deletion in the auditory nerve and stria vascularis of the cochlea than control rats [48]. Beneficial effects of CR for the prevention of ARHL has been reported in the AU/Ss, CBA/J strains of mice as well as the C57BL/6 strain, but not in the DBA/2J, WB/ReJ, or BALB/cByJ strains [57]. The effects of CR may depend on genetic background. On the other hand, a high fat diet given to Sprague Dawley rats for 12 months resulted in elevated hearing thresholds in the high-frequency region, increased ROS generation, expression of reduced nicotinamide adenine dinucleotide phosphate (NADPH) oxidase and UCP, accumulation of mtDNA common deletion, and cleaved caspase-3 and TUNEL-positive cells in the inner ear [59]. A microarray analysis study of the cochlea revealed that CR down-regulated the expression of 24 apoptotic genes, including* Bak* and BCL2-like 11 (*Bim*), suggesting that CR could prevent apoptosis of cochlear cells [58]. It has been reported that the mitochondrial deacetylase Sirtuin 3 (*Sirt3*) mediates reduction of oxidative damage and prevention of ARHL under CR [60]. CR failed to reduce oxidative DNA damage or prevent ARHL in C57B/6 mice lacking* Sirt3* [60]. In response to CR,* Sirt3* directly deacetylated and activated mitochondrial isocitrate dehydrogenase 2 (*Idh2*), leading to increased NADPH levels and an increased ratio of reduced-to-oxidized glutathione in mitochondria [60]. In cultured human kidney cells (HEK293), overexpression of Sirt3 and/or Idh2 increased NADPH levels and gave protection from oxidative stress-induced cell death [60].

## 7. Putative Role of Oxidative Stress and Mitochondrial Dysfunction in ARHL

The important role of oxidative stress and mitochondrial dysfunction in the development of ARHL has been established by reviewing previous studies. The severity of hearing loss is probably associated with cochlear degeneration. Accumulation of mtDNA damage, ROS production, and decreased antioxidant function are primarily involved in the process of cochlear senescence in response to aging stress. Mitochondria play a crucial role in the induction of intrinsic apoptosis in cochlear cells. ARHL in laboratory animals can be prevented by certain interventions, such as CR and supplementation with antioxidants. Further large clinical studies are needed to confirm whether ARHL can be prevented by the above-mentioned interventions in humans.
